# Tuning of Morphology by Chirality in Self‐Assembled Structures of Bis(Urea) Amphiphiles in Water

**DOI:** 10.1002/chem.202003403

**Published:** 2020-11-19

**Authors:** Filippo Tosi, José Augusto Berrocal, Marc C. A. Stuart, Sander J. Wezenberg, Ben L. Feringa

**Affiliations:** ^1^ Stratingh Institute for Chemistry University of Groningen Nijenborgh 4 9747 AG Groningen The Netherlands; ^2^ Groningen Biomolecular Sciences and Biotechnology Institute University of Groningen Nijenborgh 7 9747 AG Groningen The Netherlands; ^3^ Leiden Institute of Chemistry Leiden University Einsteinweg 55 2333 CC Leiden The Netherlands

**Keywords:** amphiphiles, bis(urea), chirality, ribbons, self-assembly

## Abstract

We present the synthesis and self‐assembly of a chiral bis(urea) amphiphile and show that chirality offers a remarkable level of control towards different morphologies. Upon self‐assembly in water, the molecular‐scale chiral information is translated to the mesoscopic level. Both enantiomers of the amphiphile self‐assemble into chiral twisted ribbons with opposite handedness, as supported by Cryo‐TEM and circular dichroism (CD) measurements. The system presents thermo‐responsive aggregation behavior and combined transmittance measurements, temperature‐dependent UV, CD, TEM, and micro‐differential scanning calorimetry (DSC) show that a ribbon‐to‐vesicles transition occurs upon heating. Remarkably, chirality allows easy control of morphology as the self‐assembly into distinct aggregates can be tuned by varying the enantiomeric excess of the amphiphile, giving access to flat sheets, helical ribbons, and twisted ribbons.

## Graphical abstract

**Wrapping with ribbons**: Chiral bis(urea) amphiphiles can translate their molecular scale chiral information to the mesoscopic level via self‐assembly in water. The morphology of the aggregates can be tuned by varying the enantiomeric excess of the amphiphile, giving access to flat sheets, helical ribbons, and twisted ribbons. The system presents thermo‐responsive aggregation properties, in which a ribbon‐to‐vesicles transition occurs upon heating.

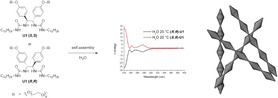

## Introduction

Self‐assembly is a very powerful bottom‐up approach to build complex architectures,[^1^, ^2^] taking advantage of information‐rich building blocks that have specific supramolecular interactions.^[3]^ The self‐assembly of amphiphilic molecules is an effective tool to generate a variety of morphologies in water.[^4^, ^5^] A plethora of self‐assembled structures has been accessed by harnessing the hydrophobic effect^[6]^ as main driving force in combination with supramolecular interactions such as π–π stacking or hydrogen bonding.^[7]^ These structures range from “simple” aggregates like micelles,^[8]^ vesicles^[9]^ and inverted structures^[10]^ to more complex architectures such as nanotubes,[^11^, ^12^, ^13^] sheets,^[14]^ and ribbons.^[15]^

Among the number of motifs available for directing self‐assembly, ureas have shown to effectively form stable soft materials. They have been largely applied as low weight molecular gelators (LWMG),[^16^, ^17^, ^18^, ^19^, ^20^] in both organic solvent[^21^, ^22^, ^23^, ^24^, ^25^, ^26^] and in water,[^27^, ^28^] as well as supramolecular polymers.[^29^, ^30^] A particularly interesting feature of the urea motif is its tendency to form highly directional intermolecular H‐bonding networks, which allows to impart high levels of order into the self‐assembled structure.[^18^, ^24^, ^31^, ^32^, ^33^, ^34^, ^35^, ^36^, ^37^, ^38^]

In an attempt to synergistically exploit intermolecular H‐bonding and the hydrophobic effect, different research groups have focused on amphiphiles containing linear and branched urea and bis(urea) motifs.[^39^, ^40^] Such structures have been explored in the formation of monolayers at the water‐air interface,^[41]^ micelles^[42]^ and rod‐like micelles.[^43^, ^44^] In certain examples, modification of the lipophilic chain of the urea‐containing surfactant led to the formation of cubic and hexagonal aggregates in aqueous medium.[^40^, ^45^] However, to the best of our knowledge, more complex architectures in water based on bis(urea) amphiphiles have not yet been discovered.

**Figure 1:**
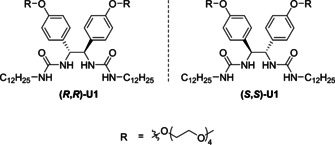
Design of bis(urea) amphiphiles (*R*,*R*)‐**U1** and (*S*,*S*)‐**U1**.

Taking the challenge on how to control the formation of more complex mesoscopic structures in aqueous medium, we designed the chiral bis(urea) amphiphile **U1**, shown in Figure 1. We envisioned that in order to tune morphologies, chirality can act as a powerful control element.[^46^, ^47^, ^48^, ^49^, ^50^, ^51^, ^52^] Tetraethylene glycol chains have been chosen as the hydrophilic component of the amphiphile to allow for good dispersion in water.[^13^, ^53^, ^54^, ^55^] Furthermore, we decided to install aliphatic chains in proximity to the bis(urea) moieties to potentially trigger the formation of a hydrogen bonding network within the hydrophobic domain of the self‐assembled structures. Herein, we show that these amphiphiles self‐assemble into thermo‐responsive chiral structures (nanoribbons) of which the morphology can be altered by heating or by simple mixing of enantiomers.

## Results and Discussion

### Synthesis and self‐assembly behavior

The bis(urea) enantiomers (*R*,*R*)‐**U1** and (*S*,*S*)‐**U1** were synthesized separately from commercially available starting materials (see the Supporting Information for synthetic details). Condensation of the corresponding enantiomer of a previously reported PEGylated diphenylethylenediamine precursor^[56]^ with 2 equivalents of dodecyl isocyanate afforded the desired products in excellent yield and optical purity [90 % yield and 98 % *ee* for (*R*,*R*)‐**U1** and 89 % yield and 96 % *ee* for (*S*,*S*)‐**U1**]. The structures were confirmed by HRMS, ^1^H NMR and ^13^C NMR spectroscopy.

**Figure 2:**
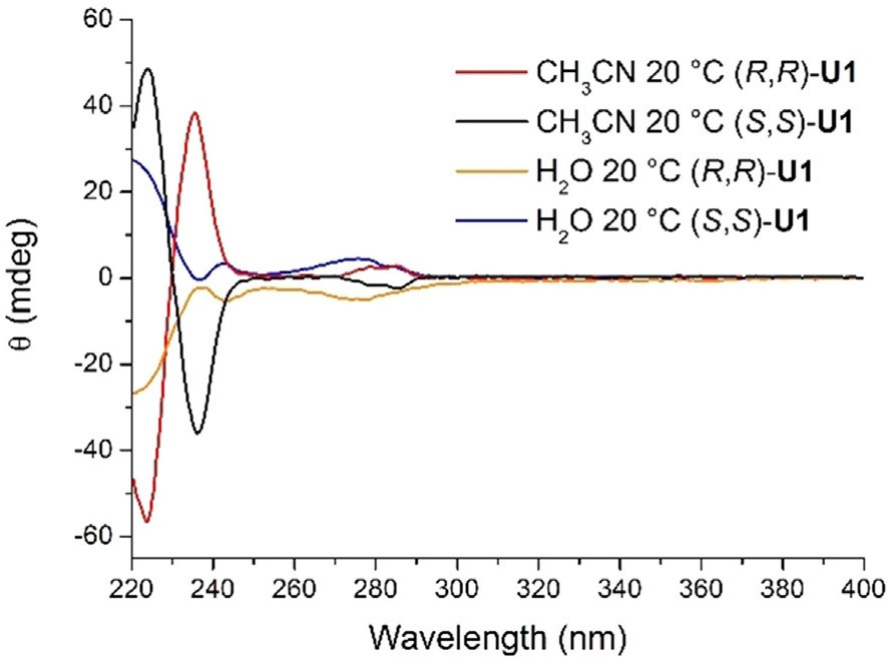
CD spectra of (*S*,*S*)‐**U1** and (*R*,*R*)‐**U1** in acetonitrile (0.5 mm) and of (*S*,*S*)‐**U1** and (*R*,*R*)‐**U1** in double distilled water (0.5 mm).

The self‐assembly behavior of these products was initially studied with circular dichroism (CD) spectroscopy (Figure 2). The CD spectrum of (*R*,*R*)‐**U1** in acetonitrile, a solvent for which ^1^H NMR dilution studies (see Figure S6 in the Supporting Information) revealed that no aggregation occurs, showed a bisignate signal at 230 nm and a positive signal at 285 nm and the exact mirror image CD spectrum was observed for (*S*,*S*)**‐U1**. When the CD spectra were recorded in water, completely different signals were observed (Figure 2). Compounds (*S*,*S*)‐**U1** and (*R*,*R*)‐**U1** presented fully positive and negative CD absorption, respectively. With respect to the spectrum recorded in acetonitrile, the signal around 230 nm was red‐shifted and much less intense (it should be noted that the lower intensity is partially due to a decrease in absorption, see Figure S9 in the Supporting Information for the UV/Vis absorption spectra). Furthermore, the signal around 275 nm displayed a broadening and a slight increase in intensity as well as a blue shift of the absorption maximum. The significantly different CD absorption in water than in acetonitrile hinted at an aqueous self‐assembly process.[^57^, ^58^]

**Figure 3:**
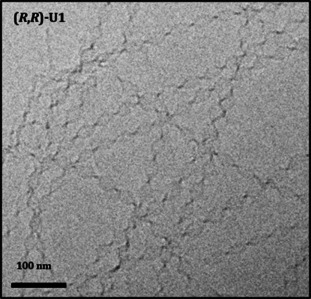
Cryo‐TEM image of twisted ribbons of (*R*,*R*)‐**U1** (2 mm in double‐distilled water).

Cryogenic transmission electron microscopy (Cryo‐TEM) confirmed the anticipated formation of self‐assembled structures in water. Both (*R*,*R*)‐**U1** and (*S*,*S*)**‐U1** gave rise to twisted ribbons (Figure 3 and Figure S14 in the Supporting Information). The structure of these ribbons was uniform within the sample, presenting a twisting pitch (for a 360° turn) of about 90 nm and a width of around 25 nm. Unfortunately, we were not able to determine the mesoscopic handedness of these ribbons, due to the bidimensional character of the Cryo‐TEM pictures.

The self‐assembled structures were further characterized by small‐angle X‐ray scattering (SAXS) experiments on (*R*,*R*)‐**U1** (see Figure S17 in the Supporting Information). The scattering profile showed a slope proportional to the inverse scattering vector square (*q* ^−2^), which is typical for flat aggregates. These experiments thus confirm the presence of the ribbon architectures observed by Cryo‐TEM.

### Thermo‐responsiveness

**Figure 4:**
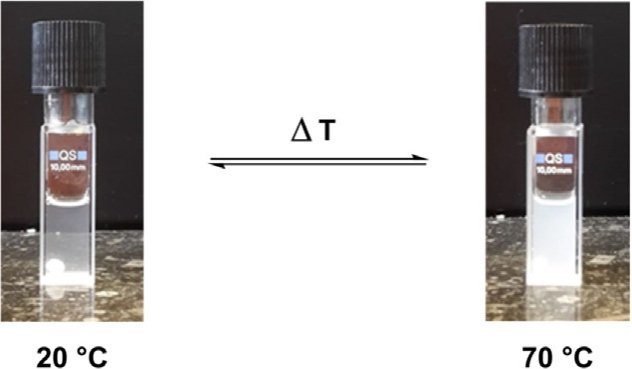
Turbidity change of a **U1** sample upon heating (1 mm).

In the sample preparation in water, an increase in turbidity was observed upon heating (Figure 4), which pointed to thermo‐responsiveness.[^59^, ^60^, ^61^, ^62^, ^63^]

Micro‐differential scanning calorimetry (micro‐DSC) provided insight into this thermo‐responsive behavior and confirmed the presence of phase transitions. In consecutive heating/cooling cycles using a 2 mm **U1**‐solution (Figure S13 in the Supporting Information), sharp transitions were observed at 33 °C (heating curve) and at 27 °C (cooling curve). These transitions were consistently detected at two different heating and cooling rates (i.e., 1 and 0.5 °C min^−1^). The thermodynamic parameters of the transitions observed upon heating and cooling are included in the Supporting Information (Figure S13). The positive values of Δ*H* and Δ*S* in the heating curve suggest that the process is entropy‐driven.

**Figure 5:**
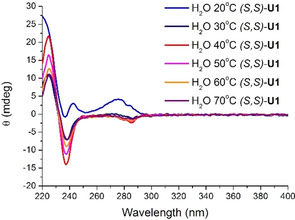
Temperature‐dependent CD spectra of (*S*,*S*)‐**U1** (0.5 mm), heating cycle.

Variable‐temperature CD measurements (between 20 and 70 °C) were then performed to further investigate the thermo‐responsiveness (Figure 5). When heating a 0.5 mm aqueous sample of (*S*,*S*)‐**U1** above 30 °C, the CD absorption spectrum resembled that of the monomeric amphiphile in acetonitrile (see Figure 2) in line with the thermal transition detected by micro‐DSC. It should be noted that a decrease in intensity of the CD signal was witnessed between 40 and 70 °C, which is due to some precipitation inside the cuvette (see the Supporting Information for details). Upon cooling of the sample, we observed an almost full recovery of the original CD absorption except for the signal at 220 nm (Figure S11 in the Supporting Information). When these experiments were repeated using (*R*,*R*)‐**U1**, comparable results were obtained (see Figure S10 in the Supporting Information). Overall, these temperature‐dependent changes in CD absorption are in line with the thermal transition observed by micro‐DSC.

**Figure 6:**
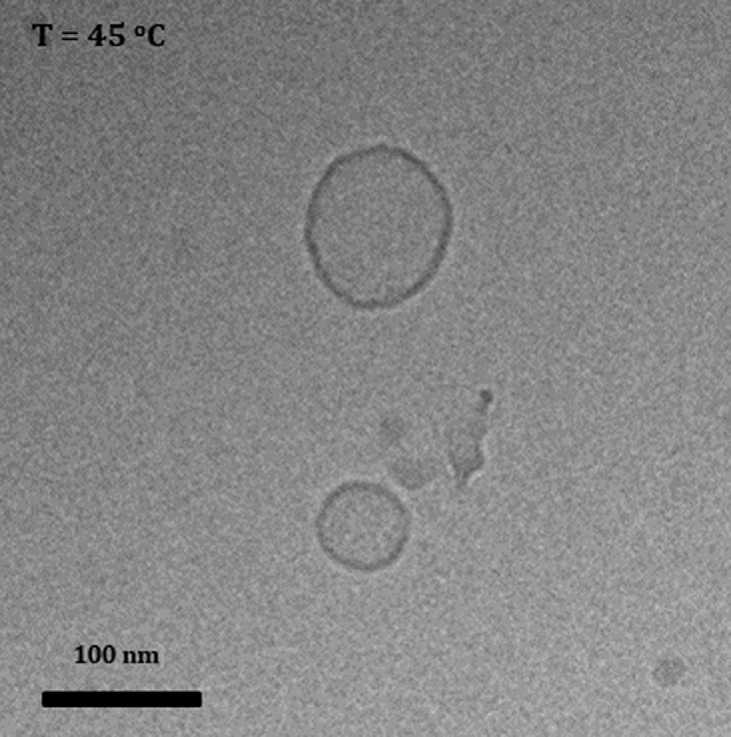
Cryo‐TEM images of self‐assembled vesicles of **U1** at 45 °C (2 mm).

Remarkably, Cryo‐TEM measurements of the heated aqueous samples revealed the formation of vesicles generated from the original twisted ribbons (Figure 6). As was also observed during the CD measurements, further beyond the thermal transition temperature, the amphiphile started to precipitate. Importantly, when the solutions were allowed to cool to room temperature, the twisted ribbons were recovered. Apparently, the vesicles have a CD spectrum that is virtually the same as that of the non‐aggregated amphiphile in the molecularly dissolved state (acetonitrile solution). Hence, above the thermal transition temperature, where vesicles form, there appears to be no translation of chirality from the molecular to the supramolecular structure, in stark contrast with the twisted ribbon formation at room temperature.

A possible explanation could be that by heating the sample, the hydrogen‐bonding interaction between amphiphiles is weakened, resulting in the formation of aggregates whose morphology depends uniquely on the hydrophobic and hydrophilic characteristics of the amphiphile, that is, vesicles. Intermolecular hydrogen‐bonding at room temperature, in the bilayer of the self‐assembled soft‐material, is therefore expected to play a role in the formation of the twisted nanoribbons, alongside the hydrophobic effect.

### Tuning morphology by mixing enantiomers

**Figure 7:**
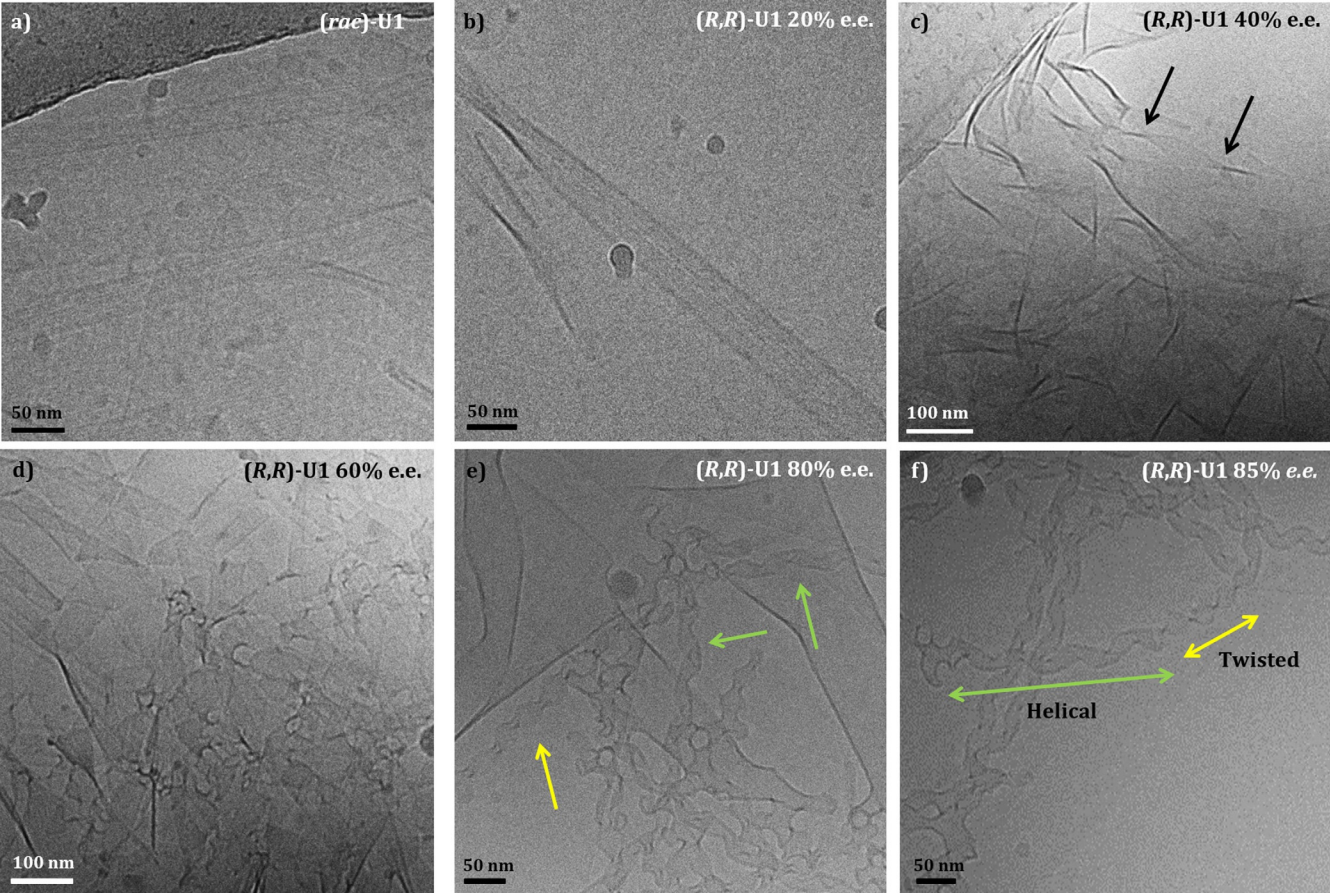
Cryo‐TEM of self‐assembled planar lamellar sheets from (a) the racemic mixture of **U1** (2 mm); (b) (*R*,*R*)‐**U1** 20 % *ee* (2 mm); (c) (*R*,*R*)‐**U1** 40 % *ee* (2 mm), arrows pointing to a twisted ribbon; (d) (*R*,*R*)‐**U1** 60 % *ee* (2 mm); (e) (*R*,*R*)‐**U1** 80 % *ee* (2 mm), green arrows pointing to helical ribbons and yellow arrows pointing to twisted ribbons; (f) (*R*,*R*)‐**U1** 85 % *ee* (2 mm), detail of a tape which includes both helical (green) and twisted (yellow) ribbon.

We finally turned our attention to investigating the aggregation behavior of (*R*,*R*)‐**U1** and (*S*,*S*)‐**U1** mixtures using Cryo‐TEM. Interestingly, the racemic mixture self‐assembled into lamellar planar sheets (Figure 7 a). The aggregate formed in this case presented a flat structure and lacked twisting as observed for the enantiomerically pure samples. The planar bilayers, on the other hand, were still comparable to the previously observed twisted ribbons in terms of packing. For a sample with an *ee* of 20 %, planar lamellar structures were still observed (Figure 7 b). At 40 % *ee* (Figure 7 c), beside planar aggregates, a few twisted ribbons were detected. These ribbons presented a twist with a pitch of around 200 nm, that is, much larger than observed for the enantiopure samples. By further increasing the *ee*, more twisted structures were observed. At 60 % *ee*, the sample was characterized by areas of coexisting planar and twisted structures (Figure 7 d). For the samples with an *ee* of 80 %, we observed a more defined situation, in which three distinct types of aggregates were present. Alongside planar sheets, helical and twisted ribbons were observed (Figure 7 e).^[57]^ For the samples having *ee* values above 80 %, we could observe ribbons presenting both a helical and a tightly twisted component, the latter comparable to the twisted ribbons observed in the highly enriched samples of **U1** (Figure 7 f).

In summary, in the case of the racemic mixture, the mesoscopic structure presents a flat architecture. By increasing the *ee*, the aggregate progressively assumes a coiled form, evident from the formation of helical and twisted tapes. Samples with high *ee* values result in areas in which twisted tapes are more frequently present and closely resemble the mesoscopic characteristics of the nearly enantiopure system. Somehow, the formation of the helical ribbons seems to be an intermediate stage, in which at much higher concentrations of one enantiomer (80 % *ee*) the flat self‐assembled structure starts to assume a coiled morphology, not quite as twisted as the one based on the pure enantiomers. We can hypothesize that the reason for this different type of twist is given by a higher local concentration of a single enantiomer in the self‐assembly process, which results in local aggregation of highly enantioenriched amphiphiles (Figure 3).

At the moment, however, we are not able to answer the question why the helical ribbon represents an intermediate state, and further investigation is needed to elucidate this phenomenon. Nevertheless, from these TEM experiments, we can conclude that the enantiomeric composition strongly influences the morphology of the aggregates.

## Conclusions

We designed a chiral bis(urea) amphiphile which self‐assembled into chiral nanoribbons in water. These nanoribbons exhibited thermo‐responsive behavior, that is, a reversible change from nanoribbon to vesicles was observed when increasing the temperature. Furthermore, by mixing the enantiomers of the amphiphile in different ratios, the outcome of the self‐assembly process could be easily changed from flat sheets to helical ribbons and twisted ribbons. Our study shows how temperature and enantiomeric composition can be used to tune the morphology of urea‐based self‐assembled materials. The new insights gained will encourage future development of stimuli‐controlled self‐assembled chiral systems in water.

## Conflict of interest

The authors declare no conflict of interest.

## Supporting information

As a service to our authors and readers, this journal provides supporting information supplied by the authors. Such materials are peer reviewed and may be re‐organized for online delivery, but are not copy‐edited or typeset. Technical support issues arising from supporting information (other than missing files) should be addressed to the authors.

Supplementary
