# Supplementary material for: Tuning of Morphology by Chirality in Self‐Assembled Structures of Bis(Urea) Amphiphiles in Water
Source: Chemistry. 2020 Nov 19;27(1):326–30. doi: 10.1002/chem.202003403 (PMC7839493; doi:10.1002/chem.202003403)
Supplement: Supplementary file 1 — Supplementary [file CHEM-27-326-s001.pdf]

Chemistry–A European Journal

Supporting Information

Tuning of Morphology by Chirality in Self-Assembled Structures of Bis(Urea) Amphiphiles in Water

Filippo Tosi,[a] José Augusto Berrocal,[a] Marc C. A. Stuart,[a, b] Sander J. Wezenberg,*[a, c] and Ben L. Feringa*[a]

Table of Contents:

1. General Experimental Procedures S2

2. Synthesis of Bis(Urea) Amphiphiles S4

3. NMR Spectra S5

4. ^{1}H NMR Dilution Experiments S8

5. HRMS Spectrum S9

6. CD and UV Spectra S10

    6.1 Temperature Dependent CD Spectroscopy S10

    6.2 LCST Measurements S11

7. Micro-DSC Diagrams S12

8. Additional Cryo-TEM Images S12

9. SAXS Measurement S13

10. Analysis of Thermal Decomposition S14

11. References S14

1. General Experimental Procedures

Materials. Dodecyl isocyanate was purchased from Sigma-Aldrich and used without further purification. Compounds ***(R,R)*-1** and ***(S,S)*-1** were synthesized following a previously reported procedure.[1] The reactions, carried out under a nitrogen atmosphere, were run using oven dried glassware and standard Schlenk techniques. CH_2Cl_2 was purified using an MBraun SPS-system. Solvents used for chromatography were of technical grade.

Chromatography. TLC was performed with Merck silica gel 60 F254 plates and visualization was accomplished by UV light and potassium permanganate staining. Flash chromatography was carried out using Merck silica gel 60 (230-400 mesh ASTM).

NMR. NMR spectra were obtained using a Varian Unity Plus Varian-400 (operating at 399.93 MHz for the ^{1}H nucleus and at 100.57 MHz for the ^{13}C nucleus). Chemical shifts are reported in δ = units (ppm), relative to the residual solvent signal of $CDCl_3$ (^{1}H NMR: δ = 7.26 ppm, ^{13}C NMR: δ = 77.0 ppm). Multiplicity is reported as follows: chemical shifts, multiplicity (s = singlet, d = doublet, t = triplet, q = quartet, br = broad, m = multiplet).

HRMS. Mass spectra were recorded on an LTQ Orbitrap XL (ESI).

UV-Vis. UV-Vis absorption spectra were measured on a Jasco V-630 spectrometer in 1 cm quartz cuvettes.

Transmittance. Transmittance measurements were carried out a Jasco V-630 spectrometer in 1 cm quartz cuvettes equipped with a micro-stirring bar. Transmittance values were recorded at 450 nm, where the amphiphilic compound did not show absorbance. Temperature scans were performed between 20 °C to 70 °C with 2 °C steps, allowing the sample to equilibrate for 5 min. LCST was evaluated as the temperature at which the transmittance of the sample reached values below 50%.

CD. CD spectra were recorded on a JASCO J-810 spectropolarimeter in 1 cm quartz cuvettes, with 4 accumulations at a scanning speed of 100 nm/min.

Optical Rotation. $[\alpha]_D^{20}$ values for ***(R,R)*-U1** and ***(S,S)*-U1** were measured in CH_3CN on a Schmidt+Haensch MH8 polarimeter in a 100 mm cell.

SAXS. Small-angle X-ray scattering experiments were performed on a SAXSLAB Ganesha system using a GeniX-Cu ultra-low divergence source producing X-ray photons with a wavelength of 1.54 Å and a flux of 1*108 ph s^{-1}. Scattering patterns were collected using a Pilatus 300K silicon pixel detector. Sample-to-detector distances of 0.73 m and 1.53 m were used giving an observed q range of 1.3*10^{-3} nm^{-1}≤ q ≤ 2.4 nm^{-1}. The solutions (0.5 mM) were measured in 2 mm quartz capillaries. The SAXSgui software package was used to radially average the resulting 2D images to obtain the intensity I(q) vs. q profiles. Standard data reduction procedures, *i.e.* subtraction of the solvent's contribution, were performed using the same software. Experimental data were fit to a lamellar form factor[2,3] using the SasView 4.1.2 software package. The scattering length density (sld) of water was set at 6.39 * 10^{-6} $Å^{-2}$, whereas the sld of the self-assembling monomer was calculated from the empirical formula and set at 7.91 * 10^{-6} $Å^{-2}$. The lamellar thickness was the only optimized parameter (optimized value = 7.23 nm). Polydispersity was not considered in the fitting process.

Micro-DSC. The measurements were taken in TA multi-cell micro differential scanning calorimeter (micro-DSC). Solutions of 1.0 mL (2 mM) were prepared following the procedures described below and transferred to the designed DSC pan for the machine, with a sealed cap. For the reference pan, 1 mL of water was used. The samples were initially cooled to 20 °C and then subjected to one heating/cooling cycle from 20 °C to 70 °C and back with a rate of 0.5 °C min^{-1}. The data presented, represents the second heating and cooling run.

Cryo-TEM. The samples were prepared by depositing a few µL of amphiphile solution on holey carbon coated grids (Quantifoil 3.5/1, Quantifoil Micro Tools, Jena, Germany). After blotting the excess liquid, the grids were vitrified in liquid ethane (Vitrobot, FEI, Eindhoven, The Netherlands) and transferred to a FEI Tecnai T20 cryo-electron microscope equipped with a Gatan model 626 cryo-stage operating at 200 keV. Micrographs were recorded under low-dose conditions with a slow-scan CCD camera. The bilayer thickness was measured on slightly defocused cryo-electron microscopy images to obtain maximal phase contrast.

Sample Preparation: 1 mL of a 2 mM solution of the compound (**(*R*,*R*)**-**U1** or **(*S*,*S*)**-**U1**) in technical grade CH_2Cl_2 or $CHCl_3$ was placed in a 4 mL vial. The solvent was slowly evaporated using a nitrogen flow and a thin film of the amphiphilic compound was formed, which was subsequently hydrated with doubly distilled water and submitted to three consecutive cycles of freeze-sonication-heat-sonication. In order to exclude possible interferences given by electrolytes or solutes, we performed the first self-assembly studies in doubly distilled water which had an Electric Conductivity (EC) of units of nS prior to use. Enantiomerically enriched mixtures of **U1** were prepared by mixing stock solutions of the separate enantiomers.

2. Synthesis of bis(urea) amphiphiles

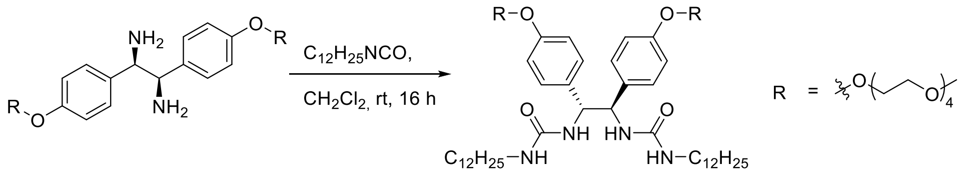

(*R*,*R*)-1 **(*R*,*R*)-U1** (90%)

Compound **(*R*,*R*)-1** (95 mg, 0.15 mmol) was dissolved in CH_2Cl_2 (10 mL) under a nitrogen atmosphere. Dodecyl isocyanate (65 mg, 74 µL, 0.31 mmol) was added and the reaction mixture was left stirring under a nitrogen atmosphere for 16 h, after which it was concentrated *in vacuo*. Purification by precipitation in 1:1:1 (v/v) CH_2Cl_2/Et_2O/Pentane mixture (6 mL) followed by column chromatography (SiO_2, CH_2Cl_2/MeOH: 99:1 → 95:5) gave pure **(*R*,*R*)-U1** (141 mg, 90%) as a white semisolid; (R_f: 0.4 CH_2Cl_2/MeOH: 95:5); ^1H NMR (400 MHz, $CDCl_3$) δ 6.87 (d, *J* = 8.7 Hz, 4H), 6.65 (d, *J* = 8.7 Hz, 4H), 6.07 (br, 2H)*, 4.80 (br, 2H)*, 4.73 (m, 2H), 4.00 (t, *J* = 4.9 Hz, 4H), 3.83 – 3.74 (m, 4H), 3.61 (m, 20H), 3.57 – 3.48 (m, 4H), 3.35 (s, 6H), 3.16 – 2.98 (m, 4H), 1.39 (m, 4H), 1.24 (m, 36H), 0.91 – 0.82 (t, *J* = 7.0 Hz, 6H); ^{13}C NMR (101 MHz, $CDCl_3$) δ 159.2, 157.8, 133.1, 128.8, 114.4, 72.2, 71.0, 70.8 (2C), 70.7, 69.9, 67.4, 60.6, 59.3, 40.8, 32.2, 30.5, 30.0 (4C), 29.7 (2C), 29.6, 27.2, 23.0, 14.4; HRMS (ESI-ion trap) *m/z*: $[M+H]^+$ Calcd for $C_{70}H_{109}N_2O_{12}$ 1047.7567, found 1047.7583; $[\alpha]_D^{20}$ = +26.74 (*c* 2.09*10^{-1}, CH_3CN).

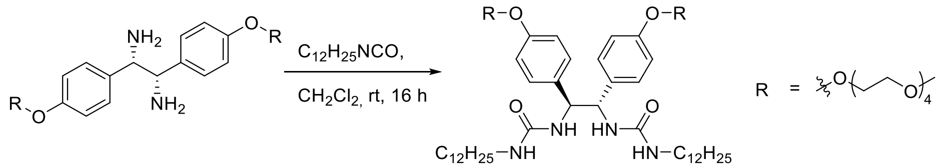

(*S*,*S*)-1 **(*S*,*S*)-U1** (90%)

Compound **(*S*,*S*)-U1** (139 mg, y = 89%) was obtained using the same procedure as for **(*R*,*R*)-U1** and the analytical data was identical, with the exception of the NH peaks (highlighted in the spectra with a *), of which the chemical shift is concentration dependent in $CDCl_3$; $[\alpha]_D^{20}$ = -25.68 (*c* 2.10*10^{-1}, CH_3CN).

3. NMR Spectra

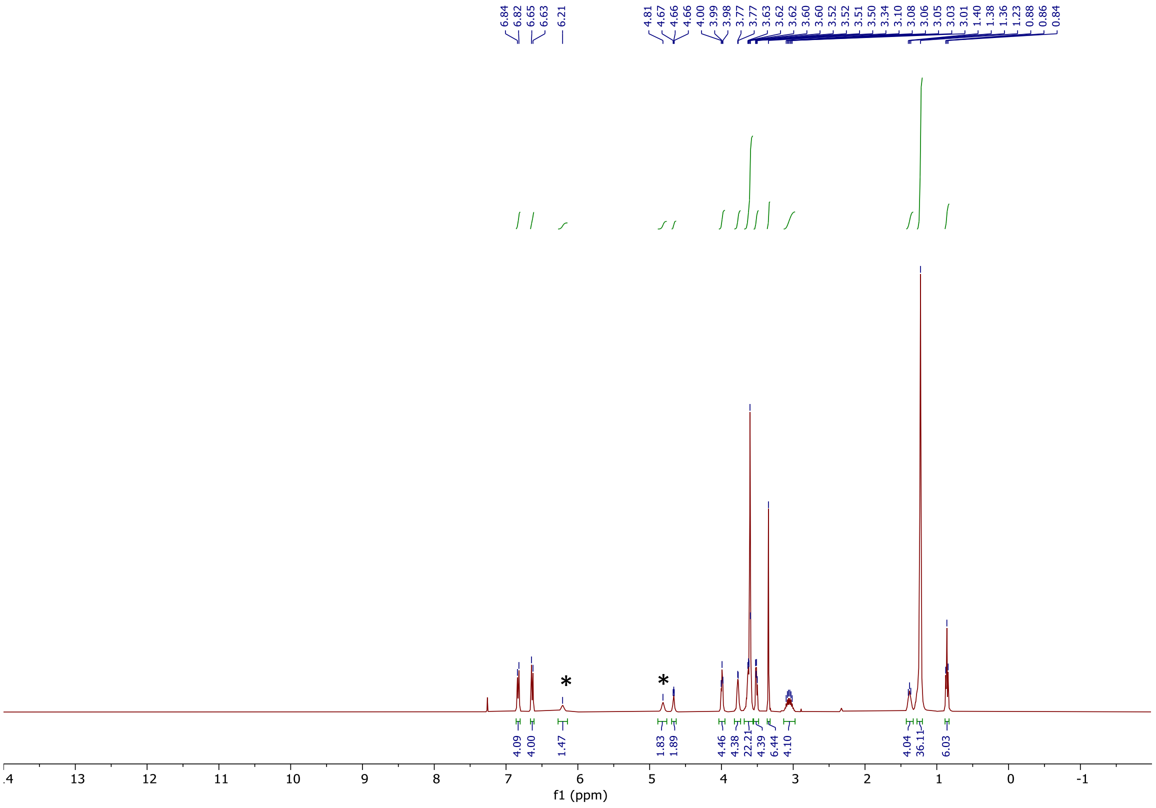

Figure S1: ^{1}H NMR of of **(*R*,*R*)-U1**, full spectrum. NH peaks are highlighted with *.

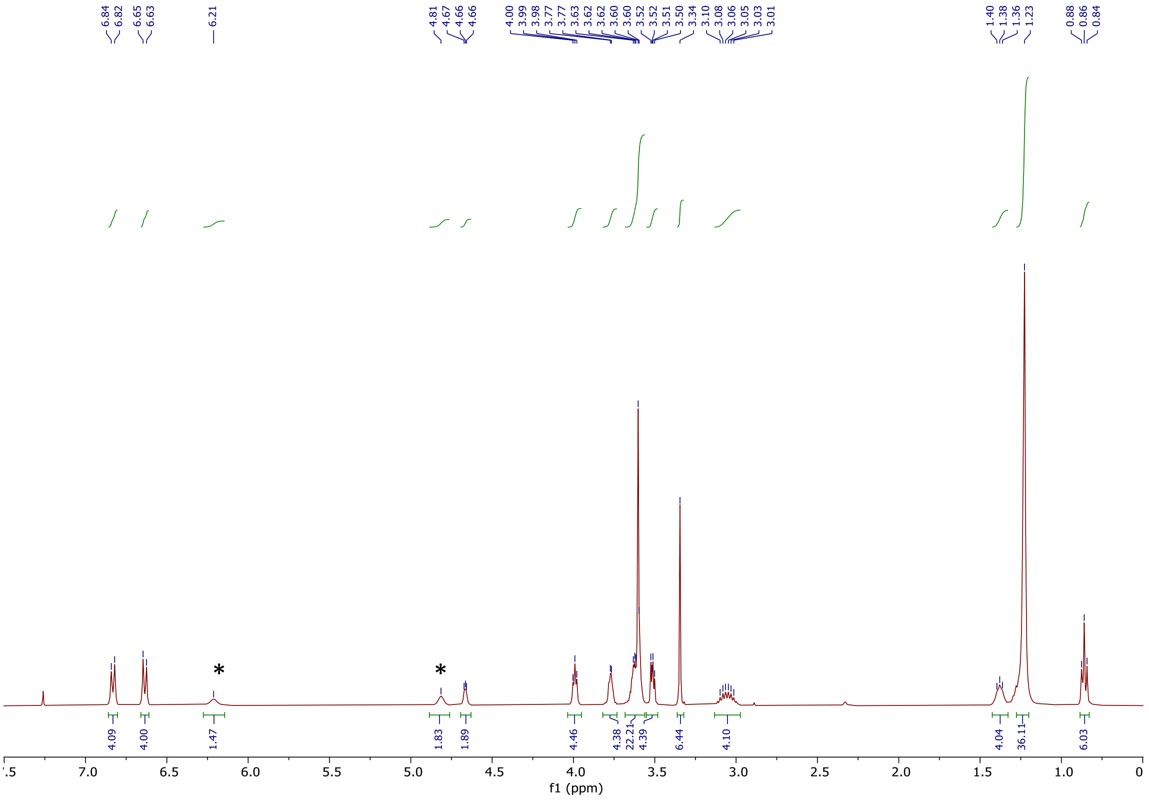

Figure S2: ^{1}H NMR of of **(*R*,*R*)-U1**, zoom 0.5 – 7.5 ppm. NH peaks are highlighted with *.

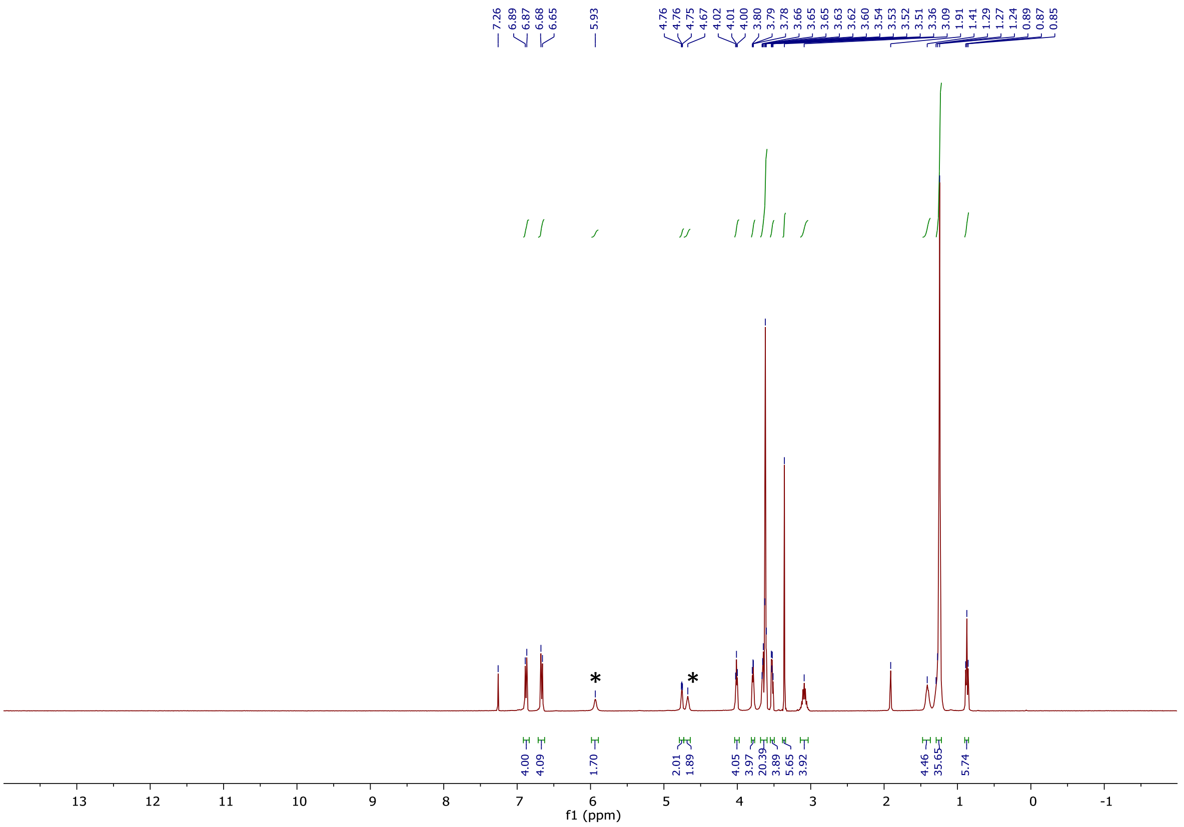

Figure S3: ^{1}H NMR of of **(*S*,*S*)-U1**, full spectrum. NH peaks are highlighted with *.

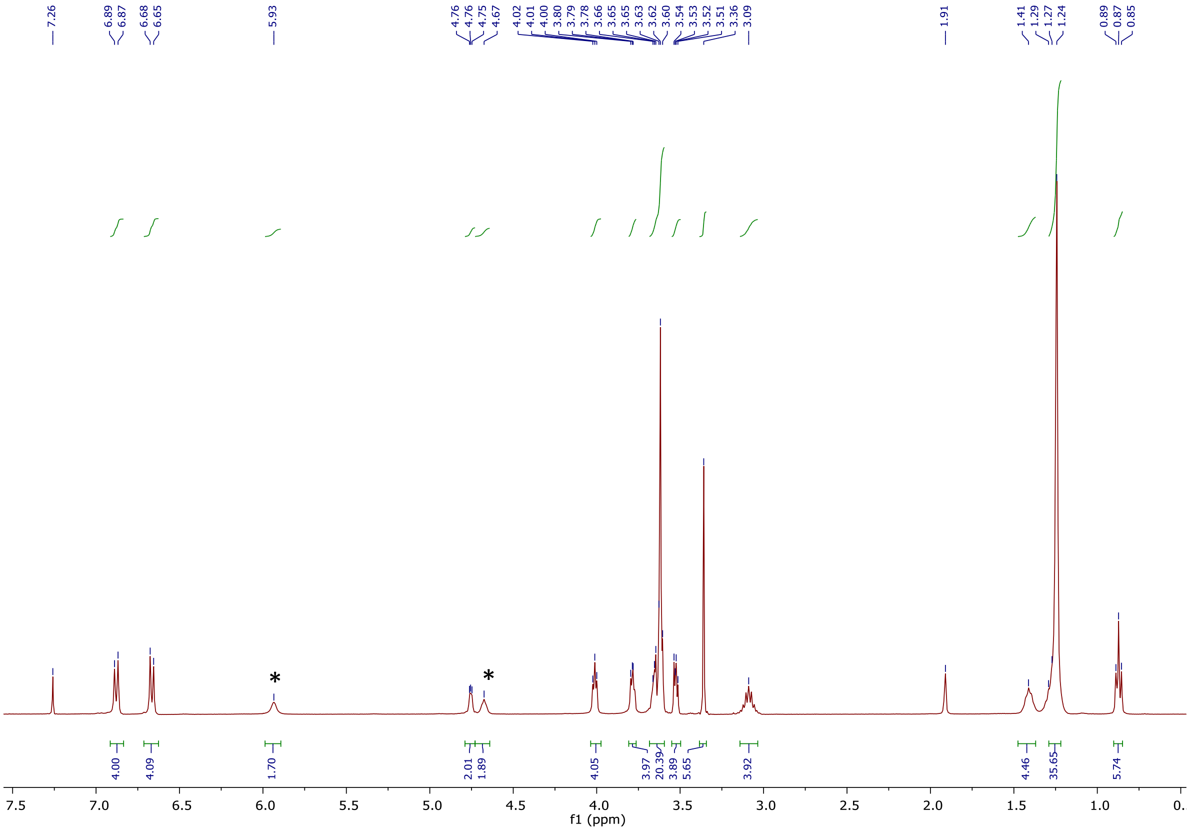

Figure S4: ^{1}H NMR of of **(*S*,*S*)-U1**, zoom 0.5 – 7.5 ppm. NH peaks are highlighted with *.

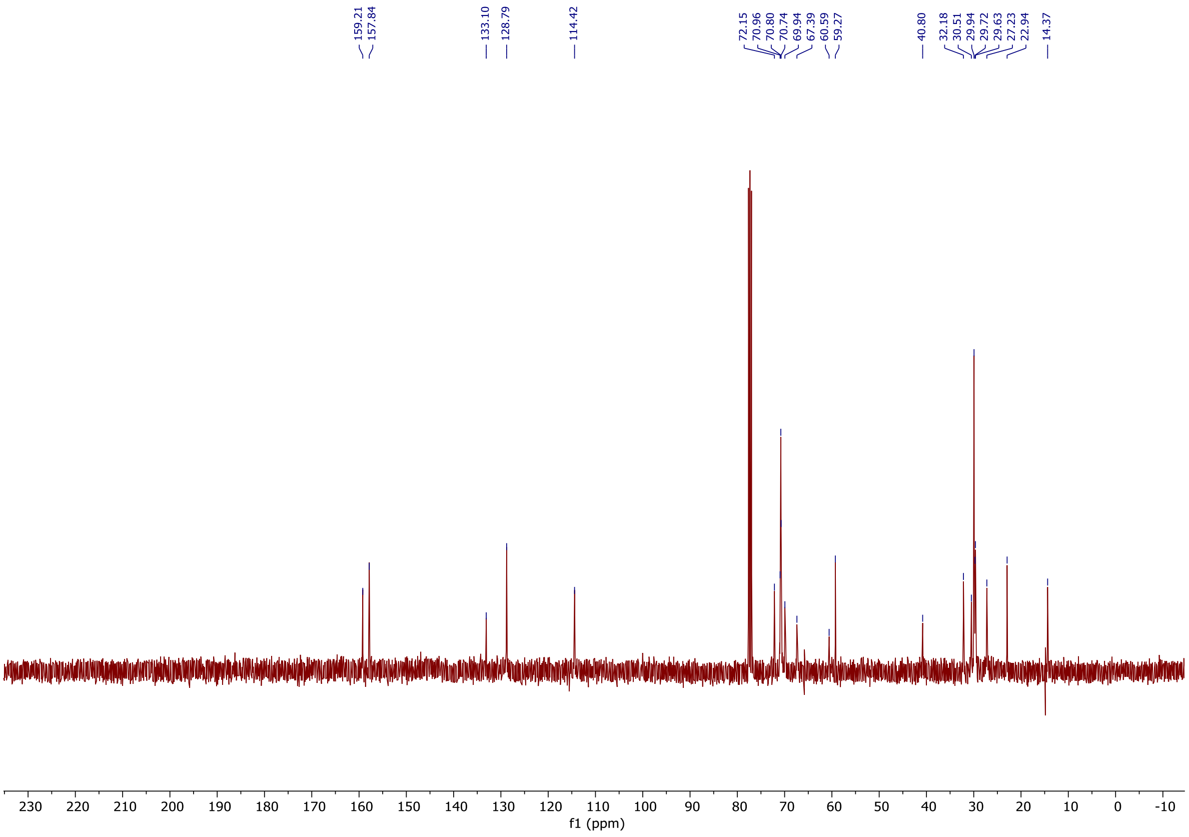

Figure S5: ^{13}C NMR of **(*R*,*R*)-U1**.

4. ^{1}H NMR Dilution Experiments

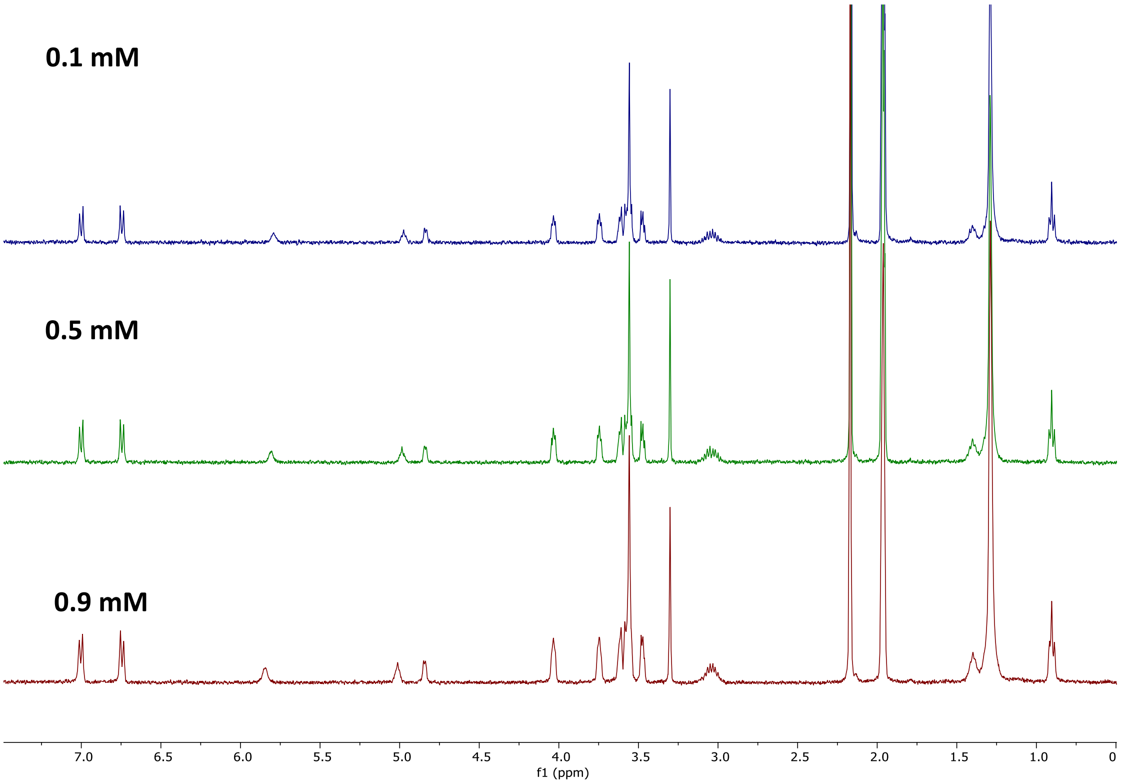

Figure S6: ^{1}H NMR spectrum of ***(R,R)*-U1** in acetonitrile-d_3 at different concentrations.

^{1}H NMR spectra were recorded at different concentrations in CD_3CN. Concentrations were chosen in a range around the sample concentration used for CD measurements. The absence of spectral changes in the ^{1}H NMR spectrum indicates that the compound does not aggregate in CH_3CN in this concentration regime.

5. HRMS Spectrum

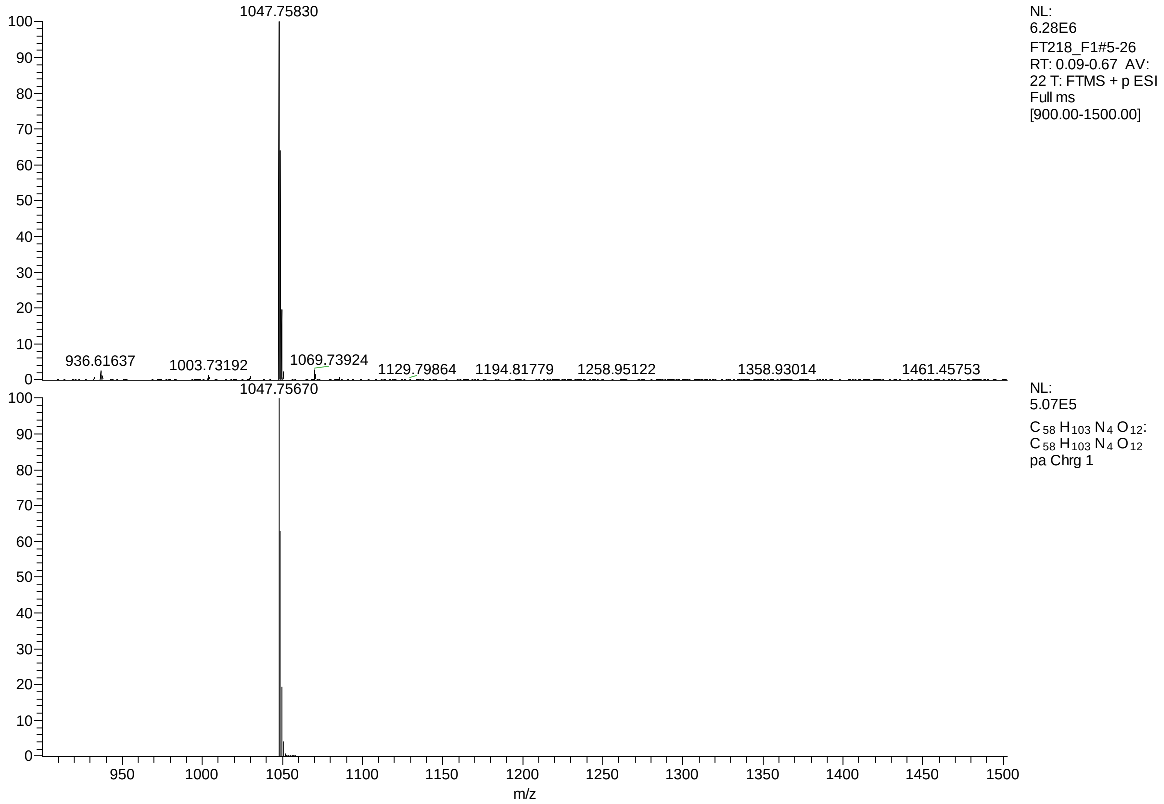

Figure S7: HRMS (ESI-ion trap) of **(*R*,*R*)-U1**; (above) experimental measurement, (below) calculated HRMS spectrum.

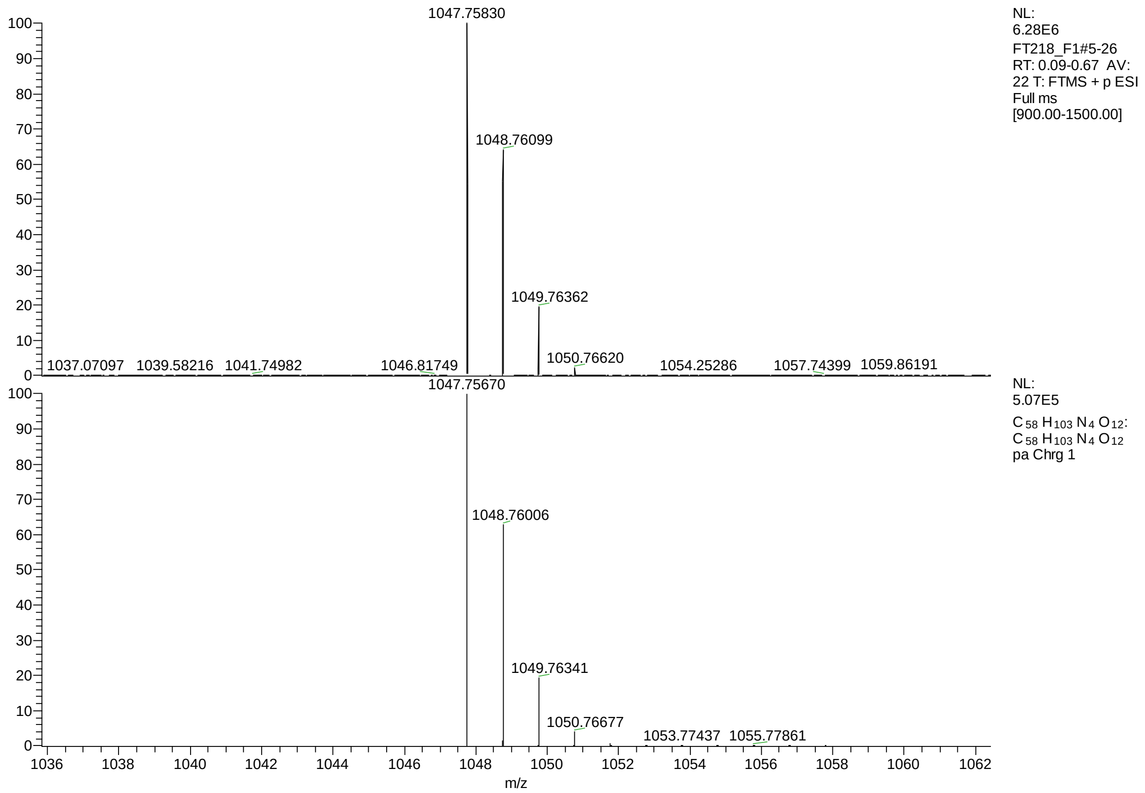

Figure S8: Zoom in HRMS (ESI-ion trap) of **U1**; (above) experimental measurement, (below) calculated HRMS spectrum.

6. CD and UV Spectra

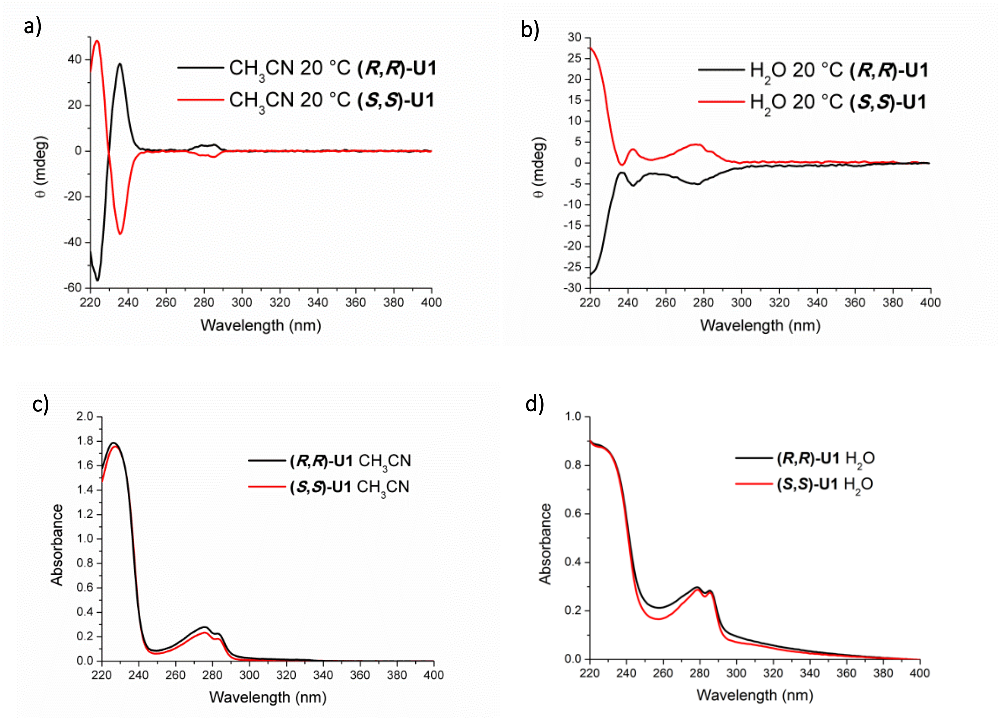

Figure S9: (a) CD spectra of **(*S*,*S*)-U1** and **(*R*,*R*)-U1** in acetonitrile (0.5 mM); (b) CD spectra of **(*S*,*S*)-U1** and **(*R*,*R*)-U1** in doubly distilled water (0.5 mM); (c) UV-Vis spectra of **(*S*,*S*)-U1** and **(*R*,*R*)-U1** in acetonitrile (0.5 mM); (d) UV-Vis spectra of **(*S*,*S*)-U1** and **(*R*,*R*)-U1** in doubly distilled water (0.5 mM).

6.1 Temperature Dependent CD Spectroscopy

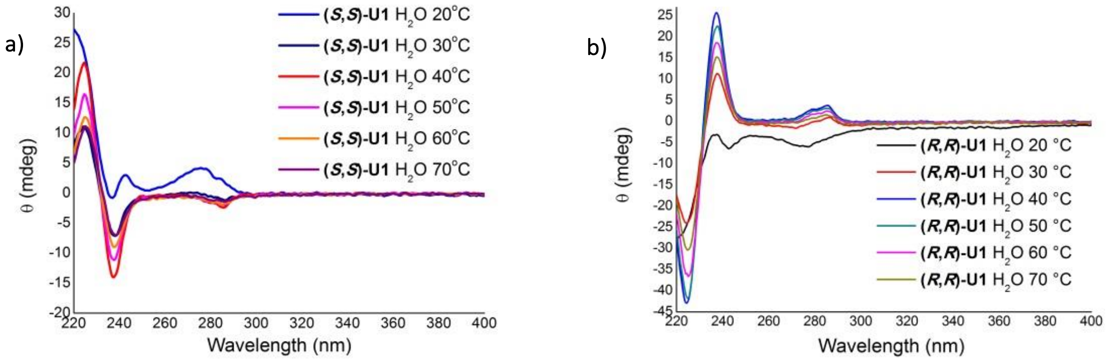

Figure S10: Temperature-dependent CD spectra of (a) **(*S*,*S*)-U1** (0.5 mM and (b) **(*R*,*R*)-U1** (0.5 mM), heating cycle.

Upon heating, the shape of the CD signal of **(*S*,*S*)-U1** changed to a signal that is similar to that of the monomeric amphiphile in solution (which is shown in Figure S7a), in correspondence with the thermal transitions observed by micro-DSC (above 30 °C). The same phenomenon is observed for amphiphile **(*R*,*R*)-U1** and the results are comparable in the spectral region between 275 and 285 nm. The same trend is observed for both amphiphiles although the intensity of the bisignate CD signal (between 200 and 245 nm) seems to be higher in the case of **(*R*,*R*)-U1** (Figure S8b), which we cannot yet explain.

The decrease in intensity of the CD signal witnessed upon heating from 40 °C to 70 °C in both cases can be explained by a degree of precipitation occurring inside the cuvette (due to the LCST of the sample).

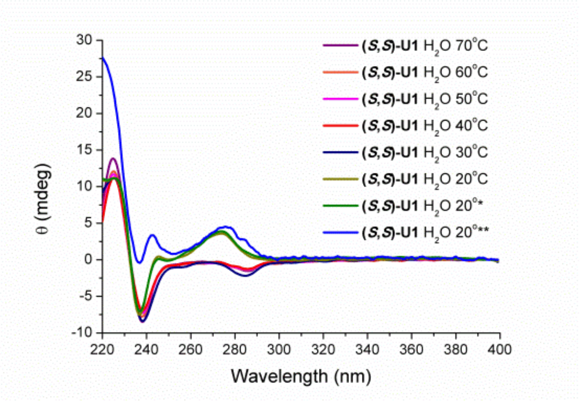

Figure S11. Temperature-dependent CD spectra of ***(S,S)*-U1** (0.5 mM), cooling cycle. (*) CD spectrum recorded in H_2O after 16 h; (**) reference CD spectrum of the starting material ***(S,S)*-U1**.

^{1}H NMR analysis of the thermally-treated sample isolated via freeze-drying and re-dissolution in $CDCl_3$ suggested partial degradation, manifested by the occurrence of additional peaks that differed from those of the starting monomer (Figure S18). However, this partial decomposition did not influence the mesoscopic nature of the ribbon over consecutive heating cycles.

6.2 LCST Measurements

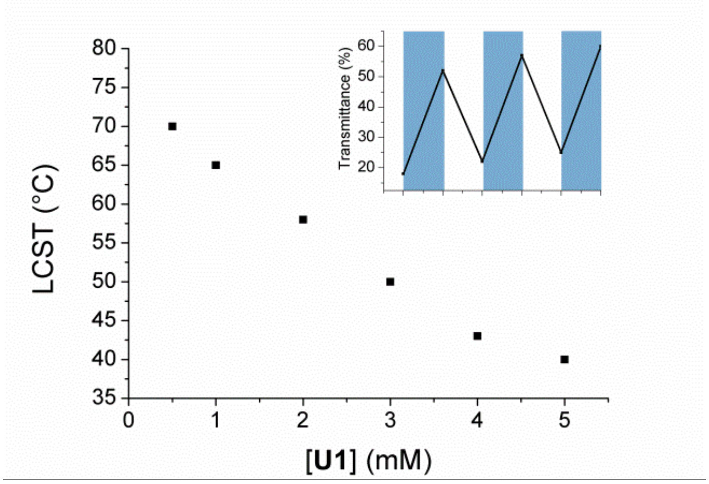

Figure S12: LCST of **U1** from 0.5 mM to 5 mM; (insert) LCST reversible behavior, heating (blue bands) and cooling (white bands).

Molecules are either in solution or in their self-assembled state below the Lower Critical Solution Temperature (LCST). However, above the LCST precipitation occurs due to weakened interactions with the aqueous medium, which is due to the ethylene glycol units expelling water molecules with increasing temperature in an entropically favourable process.[4,5] This process results in a collapse of the amphiphile, causing a change in the aggregation behaviour[6–10] and eventually precipitation.

Hence, we performed transmittance measurements to gain insight into the thermo-responsive behaviour. We measured the transmittance at 450 nm between 20 °C and 70 °C, at concentrations ranging from 0.5 to 5 mM (Figure S11). As expected, the LCST shows a linear dependence on the sample concentration. Furthermore, transmittance values were *quasi*-reversible over three consecutive heating and cooling cycles between 20 °C and 70 °C (insert Figure S11). Since the data collected in the micro-DSC experiment, CD and Cryo-TEM measurements clearly pointed towards a specific ribbon-to-vesicle transition temperature (34 °C for 2 mM sample), we hypothesize that a LCST transition possibly takes place at higher temperature.

7. Micro-DSC Diagrams

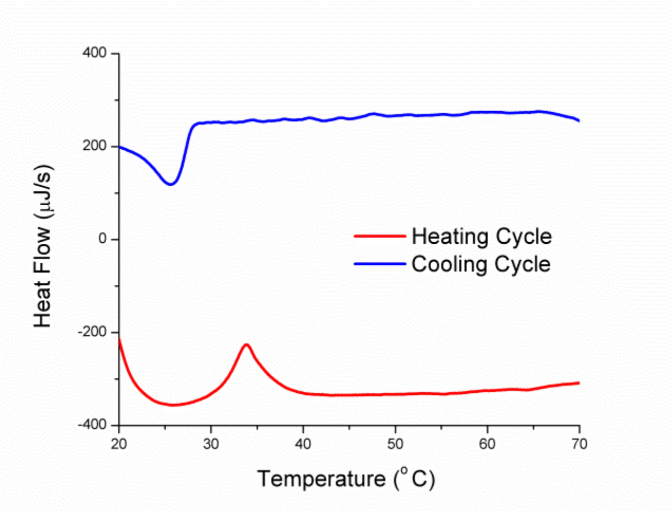

Figure S13. micro-DSC diagrams on **(*R*,*R*)-U1** (2 mM) at heating and cooling speed of 0.5 °C/min.

Thermodynamic parameters for the thermal transition observed at 33.3 ºC upon heating: ΔH = 19.8 kJ mol^{-1}, ΔS = 0.06 kJ mol^{-1} K^{-1}. Thermodynamic parameters for the thermal transition observed at 27.1 ºC upon cooling: ΔH = - 20.8 kJ mol^{-1}, ΔS = - 0.07 kJ mol^{-1} K^{-1}.

8. Additional Cryo-TEM Images

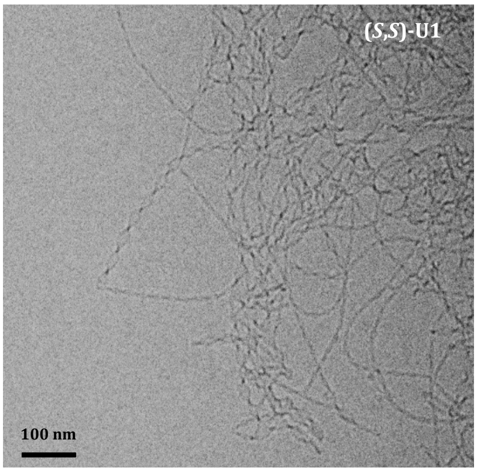

Figure S14: Cryo-TEM of twisted ribbons of **(*S*,*S*)-U1** (2 mM).

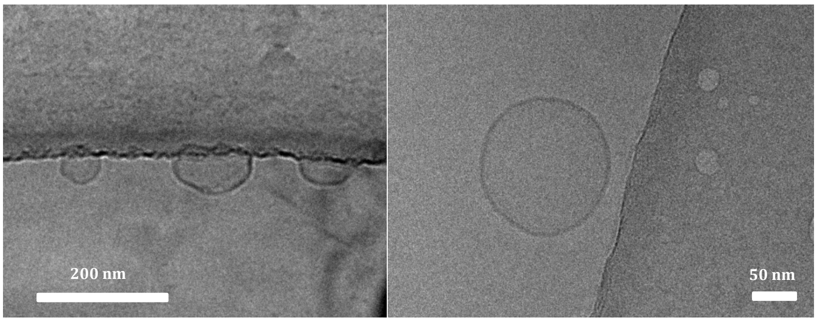

Figure S15: Cryo-TEM images of self-assembled vesicles of **U1** at 45 °C (2 mM).

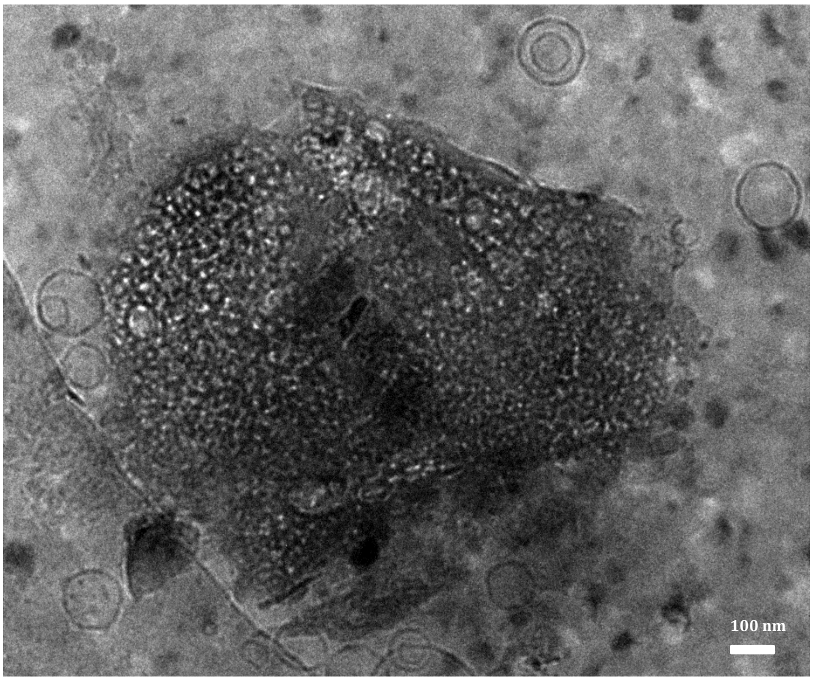

Figure S16: Cryo-TEM images of self-assembled vesicles of **U1** at 45 °C (2 mM) and precipitate.

Alongside with the formation of vesicles at the thermal transition observed with micro-DSC some precipitation occurs, also visible by Cryo-TEM (Figure S14), in agreement with the LCST data (Figure S11).

9. SAXS measurement

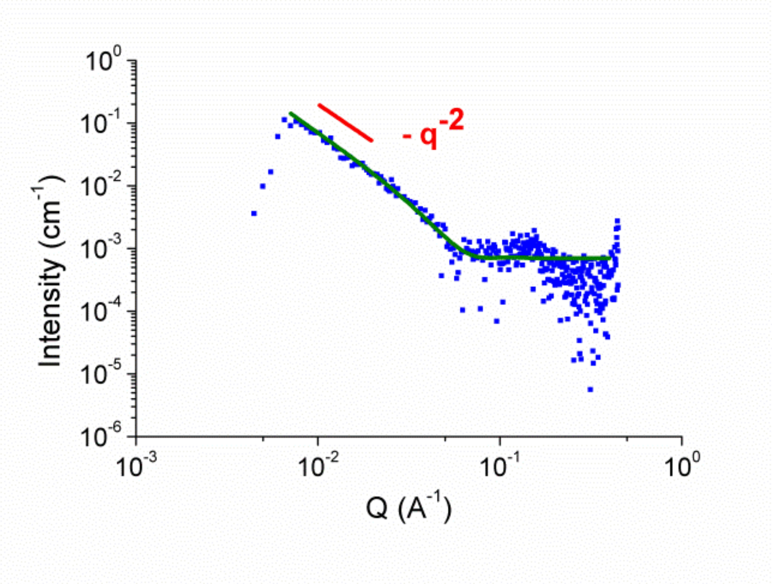

Figure S17: SAXS measurements of the twisted ribbons of **(*R*,*R*)-U1** (0.5 mM).

10. Analysis of Thermal Decomposition

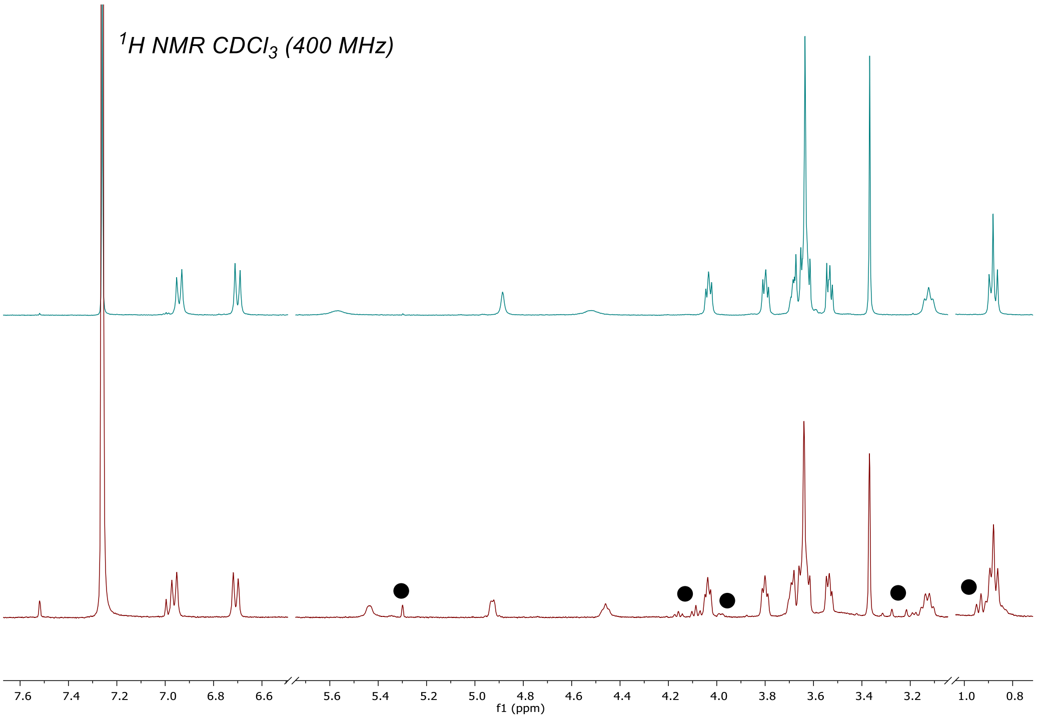

Figure S18: ^{1}H NMR ($CDCl_3$) comparison between **(*S*,*S*)-U1** before (top) and after (bottom) the heating cycles. Minor decomposition is highlighted with black dots (●).

^{1}H NMR analysis of the amphiphiles after several heating cycles showed minor decomposition. The decomposition product has not yet been identified. Attempts to characterize the decomposition product via rp-HPLC and rp-UPLC-MS were inconclusive as the amphiphile **U1** does not elute well from the column.

11. References

[1] F. Tosi, M. C. A. Stuart, S. J. Wezenberg, B. L. Feringa, *Angew. Chem. Int. Ed.* **2019**, *58*, 14935–14939.

[2] J. Berghausen, J. Zipfel, P. Lindner, W. Richtering, *J. Phys. Chem. B* **2001**, *105*, 11081–11088.

[3] F. Nallet, R. Laversanne, D. Roux, *J. Phys. II* **1993**, *3*, 487–502.

[4] M. Corti, C. Minero, V. Degiorgio, *J. Phys. Chem.* **1984**, *88*, 309–317.

[5] G. D. Smith, D. Bedrov, *J. Phys. Chem. B* **2003**, *107*, 3095–3097.

[6] J. K. Kim, E. Lee, Y. B. Lim, M. Lee, *Angew. Chem. Int. Ed.* **2008**, *47*, 4662–4666.

[7] E. Lee, Y. H. Jeong, J. K. Kim, M. Lee, *Macromolecules* **2007**, *40*, 8355–8360.

[8] E. Lee, J. K. Kim, M. Lee, *Macromol. Rapid Commun.* **2010**, *31*, 975–979.

[9] H. J. Kim, T. Kim, M. Lee, *Acc. Chem. Res.* **2011**, *44*, 72–82.

[10] Z. Huang, E. Lee, H. J. Kim, M. Lee, *Chem. Commun.* **2009**, 6819–6821.
